# Functional Connectivity Changes of Anti‐N‐Methyl‐D‐Aspartate Receptor Encephalitis: A Graph‐Theoretic and Topology‐Based Study

**DOI:** 10.1002/brb3.71145

**Published:** 2025-12-22

**Authors:** Rui Qian, Rong Guo, Yifei Li, Chenglong Li, Ling Wei, Juanjuan Zhang, Yuanyuan Guo, Yanghua Tian

**Affiliations:** ^1^ Department of Neurology The First Affiliated Hospital of Anhui Medical University Hefei China; ^2^ Department of Neurology The Second Affiliated Hospital of Anhui Medical University Hefei China; ^3^ Department of Sleep Disorders Affiliated Psychological Hospital of Anhui Medical University Hefei China; ^4^ Hefei Fourth People's Hospital Hefei China; ^5^ Anhui Mental Health Center Hefei China; ^6^ Department of Neurology The Third Affiliated Hospital of Anhui Medical University Hefei City First People's Hospital Hefei China; ^7^ Department of Psychology and Sleep Medicine The Second Affiliated Hospital of Anhui Medical University Hefei China; ^8^ The College of Mental Health and Psychological Sciences Anhui Medical University Hefei China; ^9^ Collaborative Innovation Center of Neuropsychiatric Disorders and Mental Health Hefei China; ^10^ Institute of Artificial Intelligence Hefei Comprehensive National Science Center Hefei China

**Keywords:** Anti‐N‐methyl‐D‐aspartate receptor encephalitis, Magnetic resonance imaging, Graph theory analysis, Functional connectivity, Topology

## Abstract

**Introduction:**

Anti‐N‐methyl‐D‐aspartate receptor (anti‐NMDAR) encephalitis is a type of autoimmune disorder manifesting in neurologic, cognitive, and psychological abnormalities, yet its neurological basis remains obscure. The purpose of our study was to assess the modification of functional brain networks in patients diagnosed with anti‐NMDAR encephalitis, aiming to uncover the potential pathogenic mechanisms underlying these changes and thus provide insights for further therapeutic strategies.

**Methods:**

The present study enrolled 34 healthy controls (HCs) and 35 patients suffering from anti‐NMDAR encephalitis who were subject to functional magnetic resonance imaging (fMRI) and neuropsychological testing. Topological properties and functional connectivity (FC) were analyzed using graph theory to quantify network parameters, based on the brain functional network constructed from fMRI data.

**Results:**

Compared to HCs, patients had decreased functional connectivity between most brain regions and increased connectivity only in the parietal and left frontal lobes. In contrast to the control group, patients had an increase in local efficiency (t = 2.181, *p* = 0.032) and global efficiency (t = 2.253, *p* = 0.027), and a decrease in shortest path length (t = −2.310, *p* = 0.022). In terms of regional lymph nodes, both lymph node efficiency and degree got elevated in the default mode network (DMN), whereas the subcortical network (SCN) showed enhanced efficiency and reduced intermediacy of lymph nodes.

**Conclusion:**

Our research examined the functional brain networks of anti‐NMDAR encephalitis patients and gained an understanding of the abnormalities, which underlies further investigation into neuropathophysiological mechanisms.

## Graphical abstract

1 Core Research Framework

Participants: Thirty‐five patients with anti‐N‐methyl‐D‐aspartate receptor (anti‐NMDAR) encephalitis and 34 age‐, gender‐, and education‐matched healthy controls (HCs).

Core objective: To identify abnormalities in whole‐brain functional network topological properties and functional connectivity (FC) in patients with anti‐NMDAR encephalitis using graph‐theoretic analysis, and to elucidate the neuropathological mechanisms of the disease.

2 Technical Pipeline

Data acquisition: Resting‐state blood oxygen level‐dependent (BOLD) functional magnetic resonance imaging (fMRI) data were acquired using a 3.0T MR scanner. Concurrent neuropsychological assessments included the Mini‐Mental State Examination (MMSE), Hamilton Anxiety Scale (HAMA), and Hamilton Depression Scale (HAMD).

Network construction: Nodes were defined based on the Dosenbach 160‐region atlas (142 regions of interest [ROIs] retained after excluding 18 cerebellar ROIs). Pearson correlation analysis was used to calculate the correlation of BOLD signals between node pairs, followed by Fisher z‐transformation to generate the functional connectivity matrix.

Analytical methods: Edge‐based FC analysis: Network‐Based Statistic (NBS) was applied to compare inter‐group differences in 21 inter‐network and 7 intra‐network connections.

Graph‐theoretic analysis: Quantitative assessment of global topological parameters (e.g., global efficiency [Eglob], local efficiency [Eloc], and shortest path length [Lp]) and nodal parameters (e.g., nodal efficiency, nodal degree, and nodal betweenness).

Statistical validation: Two‐sample t‐tests (adjusted for age, gender, and years of education) and chi‐squared tests were used to analyze inter‐group differences.

3 Key Results

Abnormal functional connectivity: Patients exhibited significantly decreased FC in most brain regions (e.g., between the default mode network [DMN] and somatosensory‐motor network [SMN]). Increased FC was only observed in the parietal‐left frontal lobe (between the dorsal attention network [DAN] and SMN) and within the SMN (frontal‐precentral gyrus, precentral gyrus‐parietal lobe).

Altered global topology: Compared with HCs, patients showed increased Eglob (t = 2.181, *p* = 0.032) and Eloc (t = 2.253, *p* = 0.027), and shortened Lp (t = −2.310, *p* = 0.022), indicating abnormally enhanced global information integration and shortened information transmission paths in the brain network.

Nodal‐level abnormalities: Increased nodal efficiency and nodal degree in the DMN (ventral prefrontal cortex and frontal lobe) and SMN (precentral gyrus). Increased nodal efficiency in the insula (ventral attention network [VAN]). Decreased nodal betweenness but increased nodal efficiency in the subcortical network (SCN and thalamus).

4 Scientific Significance

This study is the first to systematically reveal the “high integration‐short path” abnormal topological pattern of whole‐brain functional networks in patients with anti‐NMDAR encephalitis. It identifies specific connectivity disruptions in core networks (e.g., DMN, SMN, and SCN), providing neuroimaging evidence for understanding the neuropathological mechanisms of the disease (e.g., NMDAR‐mediated synaptic dysfunction). These findings offer novel insights for clinical diagnosis and the development of therapeutic targets.

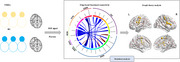

## 1 Introduction

Anti‐N‐methyl‐D‐aspartate receptor (anti‐NMDAR) encephalitis appears to be connected with a generation of autoantibodies toward the GluN1 substrate of NMDAR (Dalmau et al. 2008). It accounts for 80% of all autoimmune encephalitis (Dalmau and Graus 2018), and its morbidity in the last decade has been on the rise (Ren et al. 2021). Autoimmune antibodies bind and inactivate NMDAR and may induce NMDAR‐mediated synaptic loss and corresponding neurological deficits (Huang et al. 2020). Anti‐NMDAR encephalitis patients typically exhibit a wide range of neuropsychiatric conditions, such as dyskinesias, epileptic seizures, autonomic instability, cognitive deficits, consciousness disorders, central hypoventilation, and psychosis (Dalmau et al. 2008). Though acute clinical conditions can occur in individuals suffering from NMDAR‐resistant encephalitis, most show normal findings on conventional MRI (Graus et al. 2016; Heine et al. 2015), leading to the clinical radiology paradox. Nevertheless, previous studies in the literature have found abnormalities of brain function among anti‐NMDAR encephalitis patients. Functional magnetic resonance imaging (fMRI) and diffusion tensor imaging reveal both structural and functional alterations across various brain regions of individuals diagnosed with anti‐NMDAR encephalitis (Cai et al. 2020). Resting state fMRI, grounded on blood oxygen level‐dependent (BOLD) contrast, permits the investigation of intricate neurologic pathways to the functional detachment and integration of the brain (Barkhof et al. 2014), and as observed in anti‐NMDAR encephalitis patients, abnormalities in their interregional functional connectivity (FC) are correlated with disease duration, severity, and cognitive function (Finke et al. 2013; Peer et al. 2017). Voxel‐based morphometric analysis revealed that atrophy of NMDAR‐resistant encephalitis patients in gray matter of the thalamus was concentrated in the frontal and temporal lobes (Long et al. 2022). ^18^F‐fluorodeoxyglucose (^18^F‐FDG) metabolic network analysis by positron emission tomography (PET) provides information on functional interregional connectivity. Common abnormalities reported cover metabolic hyperactivity in the insula, temporal, and frontal lobes, with the parietal and occipital lobes hypothesized (Bacchi et al. 2018; Kerik‐Rotenberg et al. 2020). Anti‐NMDAR encephalitis not only correlated with compromised regional junctions within isolated neural loops (Finke et al. 2013) but also spanned various decentralized systems of the brain (Peer et al. 2017), which implied anti‐NMDAR encephalitis as a worldwide disease of network dysfunction.

Functional brain networks demonstrate a range of topological features intermediate between randomness and regularity (Achard et al. 2006; He et al. 2007), which contains hubs, modularity, and efficient small‐worldness (Rocchi et al. 2020), by dissociating and combining processing of information, enabling information processing and thought representation (Bullmore and Sporns 2009). There is growing evidence that all sorts of brain illnesses are related to the particular interference of these allocations (Wang et al. 2020). Previous studies point to FC reflecting statistical interdependence of brain activity (Li et al. 2025; Wang et al. 2021), and modifications in clinical mental status rely on the coordination of information flow between the distinct functional networks. Graph theory analysis offers unique advantages in exploring brain network changes in this disease. It abstracts the brain into a model composed of nodes (brain regions) and edges (FCs), enabling quantitative measurement of key topological indicators such as global efficiency, local efficiency, and shortest path length. These indicators can intuitively reflect the brain network's ability to integrate information globally and segregate functions locally (Bassett and Bullmore 2009; Bullmore and Sporns 2009). Compared with traditional functional imaging analysis methods (e.g., regional homogeneity and seed‐based functional connectivity), graph theory is more suitable for investigating the overall organizational mode of the whole‐brain network rather than focusing on individual brain regions or local connections. It can reveal abnormal network characteristics (e.g., changes in small‐world properties) that are difficult to detect with traditional methods, thereby providing a more comprehensive perspective for understanding the global network dysfunction caused by anti‐NMDAR encephalitis.

Most prior studies have primarily focused on alterations in local brain regions or isolated functional connections, lacking a systematic exploration of the topological properties of the whole‐brain functional network and failing to integrate graph theory to deeply dissect the abnormal patterns of network integration and segregation. In contrast to these prior efforts, our study moves beyond a narrow focus on local or isolated connections and systematically analyzes the topological properties of the whole‐brain functional network at both the global and nodal levels based on graph theory. Furthermore, we integrate edge‐based functional connectivity comparisons with network parcellation schemes (e.g., default mode network, subcortical network) to clarify the abnormal interaction patterns across distinct brain networks. Collectively, this study aims to identify specific alterations in the structural or functional connections of the brain in patients with anti‐NMDARencephalitis.

## 2 Methods

### 2.1 Participants

We enrolled 35 anti‐NMDAR encephalitis patients after the acute phase of the disease attending a tertiary care hospital in Hefei, China. Standards with respect to the diagnosis of anti‐NMDAR encephalitis were fulfilled in all patients, which comprised a positive immunoglobulin G NMDAR antibody detection in the cerebrospinal fluid and typical clinical signs (Dalmau et al. 2011). Elimination standards of this study comprised pregnancy, substance abuse, neurological illness, life‐threatening somatic disorders, and additional comorbid psychiatry impairments. We also excluded patients with comorbid autoimmune diseases (including systemic lupus erythematosus, Sjögren's syndrome, rheumatoid arthritis, multiple sclerosis, and myasthenia gravis) via detailed medical history collection, physical examinations, and laboratory tests for systemic autoimmune serological markers (e.g., antinuclear antibody, anti‐double‐stranded DNA antibody, and anti‐Sjögren's syndrome A/B antibodies). Thirty‐four healthy controls with education, age, and sex matched to patients, who had no mental or neurological conditions, participated in our study and tested negative for anti‐NMDAR antibodies. All subjects were free of MRI prohibitions. Written informed consent was obtained from all participants, and the study was approved by the Ethics Committee of Anhui Medical University.

### 2.2 Neuropsychological and Clinical Evaluation

The below neuropsychological assessments: (1) Hamilton Depression Scale (HAMD) and Hamilton Anxiety Scale (HAMA) to evaluate psychological well‐being and mood conditions (Hamilton 1959; Wang et al. 2012) and (2) Mini‐Mental State Examination (MMSE) (Nasreddine et al. 2005) was administered to all participants to evaluate mental health and emotional state.

### 2.3 MRI Data Acquirement and Preprocessing

All MRI scanning was performed with a GE 3.0T MR scanner (Discovery 750; GE Healthcare, Milwaukee, WI) at the University of Science and Technology of China, consisting of T1‐weighted MRI and fMRI of BOLD. All participants were directed to remain awake, loosen, cover their eyes, minimize their motions, and think nothing special during the scan. Sagittal T1‐weighted images using 3D brain volume sequence parameters were as follows: slice thickness = 1 mm, echo time (TE) = 3.18 ms, field of view (FOV) = 256 mm × 256 mm, acquisition time = 283 s, repetition time (TR) = 8.16 ms, inversion time = 450 ms, matrix size = 256 × 256, flip angle (FA) = 12°, voxel size = 1 × 1 × 1 mm, and 188 slices. Pre‐processing of resting‐state fMRI samples comprises the steps described below. To equilibrate the magnetization, we discarded the first 10 volumes. Slice timing correction and realignment were then performed. In this study, all head displacements and rotations were less than 3 mm or 3°. Then, all data were standardized to the Montreal Neurological Institute template of 3 × 3 × 3 cubic millimeter resolution and processed for spatial smoothing with a Gaussian kernel at six millimeters half‐maximum full width. Finally, regression was performed on both signals in cerebrospinal fluid and white matter, and time zone routes (0.01–0.1 Hz) filtering was applied to all models.

### 2.4 Brain Functional Network Construction

Definitions for brain nodules were in accordance with Dosenbach's 160 atlases (Dosenbach et al. 2010). Between cerebral knots, FC determined the brain margins, and the edges between nodes and nodes form the topological network. As not all available scans completely overlap the cerebellum, removing its 18 regions of interest (ROIs), there were 142 ROIs. A sphere of radius 5 mm for each node was generated centered on the map co‐ordinates. The neural signals were then drawn for each node by performing an averaging across the preprocessed BOLD signals from every element in the sphere. We calculated the Pearson coefficient of correlation in BOLD signaling among all node pairs and then Fisher‐converted them to z‐values to produce the connection matrix for the brain.

### 2.5 Edge‐Based Functional Connectivity Comparison

Taking the Network‐Based Statistic (NBS, https://www.nitrc.org/projects/nbs) methodology, we initially compared edge‐based FCs between HCs and the anti‐NMDAR encephalitis patients. NBS can offer more statistical capacity than large‐scale mono‐variate analyses (Zalesky et al. 2010). To better characterize the critical clusters gained from the NBS‐based contrasts, we additionally categorized super threshold margins based on their belonging to the network identified in Yeo et al. (2011). We applied subcortical networks rather, as edge networks encompass little ROIs. The 7 networks appear as the visual network (VN, 22 ROIs in the posterior fusiform gyrus and occipital lobe), somatosensory‐motor network (SMN, 29 ROIs located in the auditory cortex and precentral and post central gyrus), dorsal attention network (DAN, 14 ROIs in the angular gyrus, premotor cortex, superior parietal lobule and temporo‐occipital cortex), ventral attention network (VAN, 16 ROIs located in the supplementary motor area, supramarginal gyrus, insula and middle frontal gyrus), subcortical network (SCN, 7 ROIs in the thalamus and putamen), frontoparietal network (FPN, 21 ROIs located in the precuneus, lateral frontal cortex, dorsal cingulate cortex and superior parietal lobule), and default mode network (DMN, 33 ROIs located in the ventral and medial prefrontal cortex, inferior parietal lobule, posterior cingulate cortex and lateral temporal cortex). We calculated the amounts of edges attributable to the 21 inter‐network categories and the 7 intra‐network classes, respectively.

### 2.6 Graph Theory‐Based Network Analyses

In order to ascertain the variation within nodal and global network topological features during anti‐NMDAR encephalitis, we evaluated the nodal parameters (nodal betweenness, nodal degree, and nodal efficiency) and global parameters, including global efficiency (Eglob), clustering coefficients (Cp), normalized shortest path length (λ), local efficiency (Eloc), shortest path length (Lp), normalized clustering coefficient (γ), and small‐world coefficient (σ) (Watts and Strogatz 1998). By the time it displayed a bigger γ and an almost λ compared to a stochastic network of matches, the network showed small‐world property. Sparsity for the sophisticated network analysis extended from 0.1 to 0.5 at intervals of 0.01, and area under the curve (AUC) values for universal and node networks parameters between 0.1 and 0.34 were calculated for statistical analysis.

### 2.7 Statistical Analysis

Two‐sample t‐tests and chi‐squared tests were employed to detect discrepancies between HCs and anti‐NMDAR encephalitis patients on overall and nodal indicators in statistical demographic and clinical aspects at baseline. To explore topological properties of two groups via two‐sample t‐tests with correction for age, gender, and education years.

## 3 Results

### 3.1 Demographic and Clinical Characteristics

The ultimate analysis covered 35 patients with anti‐NMDAR encephalitis (17 females, 18 males) and 34 healthy controls (9 females, 25 males). No obvious intergroup variations in age (t = −0.911, *p* = 0.366), education level (t = −1.681, *p* = 0.097) and sex ratio (χ^2^ = −1.917, *p* = 0.060) were noted. Patients with anti‐NMDAR encephalitis had higher MMSE scores, HAMA scores, and HAMD scores than the HCs (*p* < 0.001). See Table 1 for demographic figures.

**TABLE 1:** Demographic and clinical characteristics of the anti‐NMDAR encephalitis patients and HC groups.

	Anti‐NMDAR encephalitis patients	Healthy controls	t/χ^2^ value	*p* value
Sample size	35	34		
Age (years)	30.11 ± 12.78	32.85 ± 12.17	−0.911	0.366
Sex (female, %)	18(51.43%)	25(73.53%)	−1.917	0.060
Education (years)	11.43 ± 3.51	13.18 ± 5.02	−1.681	0.097
MMSE scores	27.29 ± 2.19	29.50 ± 1.21	−5.174	< 0.001
HAMA scores	4.66 ± 4.96	1.94 ± 2.12	2.941	< 0.001
HAMD scores	3.17 ± 4.96	1.56 ± 1.74	1.792	< 0.001

Abbreviations: anti‐NMDAR = anti‐N‐methyl‐D‐aspartate receptor, HC = healthy control, MMSE = Mini‐Mental State Examination, HAMA = Hamilton Anxiety Scale, HAMD = Hamilton Depression Scale.

### 3.2 Edge‐Based Functional Connectivity

**FIGURE 1:**
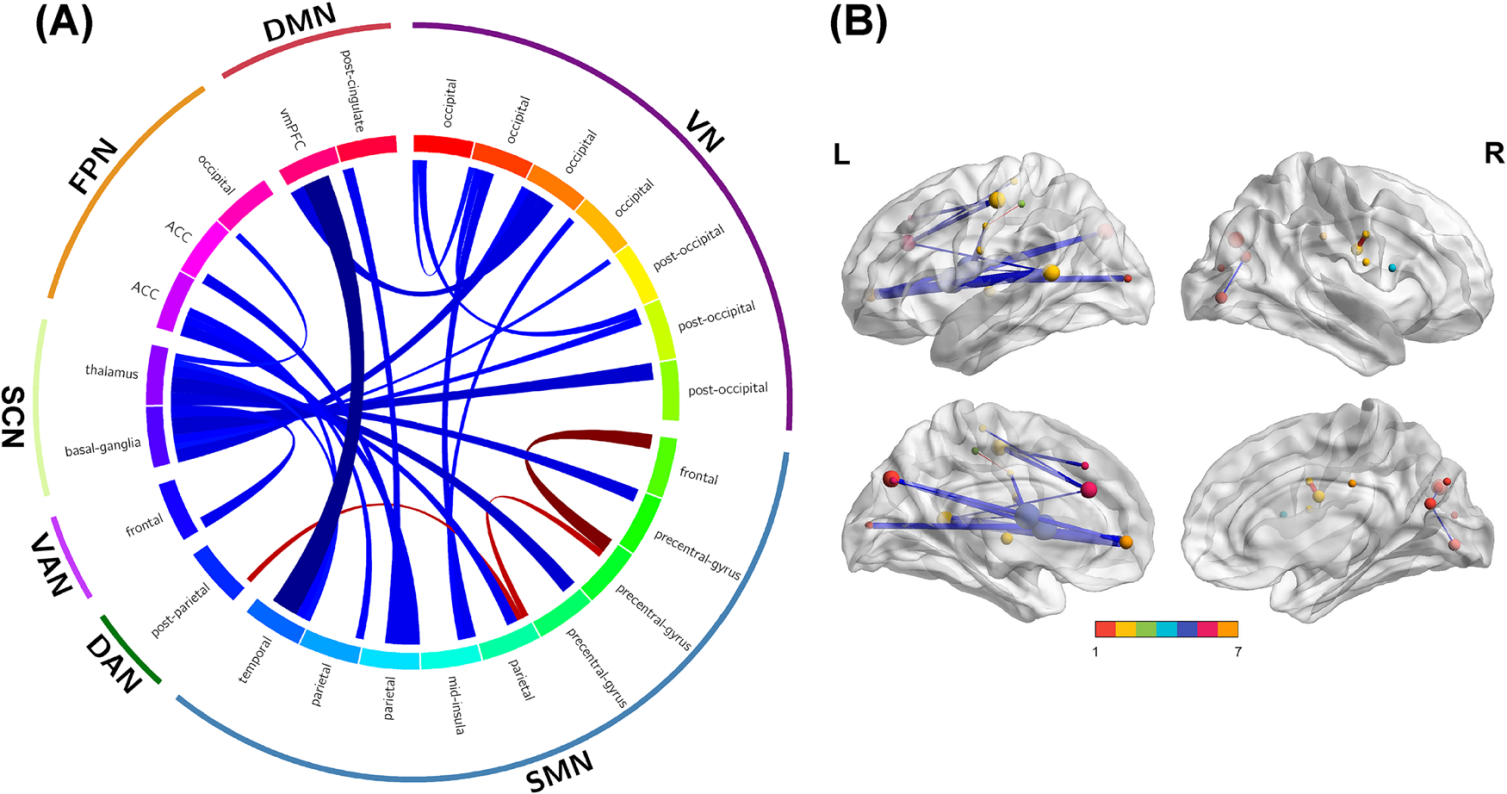
Functional connectivity decreased on a large‐scale among anti‐NMDAR encephalitis patients compared with healthy individuals. Defined the coordinates of each brain area based on the Dosenbach's 160 atlas, figure showed the same or different patterns of functional connectivity in anti‐NMDAR encephalitis patients and healthy controls. The blue line indicates a decrease in functional connectivity in patients with anti‐NMDAR encephalitis compared to healthy controls, while the red line indicates an increase. The color bar indicates seven networks, and 1 to 7 are representated as VN, SMN, DAN, VAN, SC, FPN, and DMN. Abbreviations: anti‐NMDAR = anti‐N‐methyl‐D‐aspartate receptor, DAN = dorsal attention network, DMN = default mode network, FPN = frontoparietal network, HCs = healthy controls, SCN = subcortical network, SMN = somatosensory‐motor network, VAN = ventral attention network, VN = visual network.

Dramatic discrepancies in 25 FCs were present in patients with anti‐NMDAR encephalitis versus HCs. Most of these FCs noticeably declined among anti‐NMDAR encephalitis patients, especially DMN to SMN, nevertheless, a minority of FCs were markedly enhanced, like from SMN to DAN (parietal lobe to left frontal lobe) and within SMN (frontal to precentral‐gyrus, precentral‐gyrus to parietal) (Figure 1).

### 3.3 Network Topological Characteristics

**FIGURE 2:**
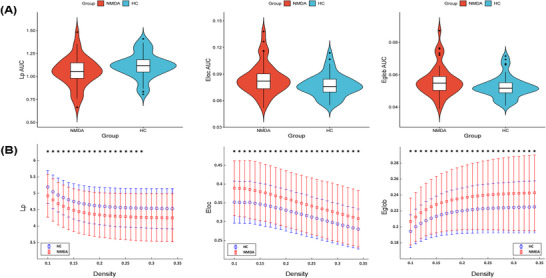
Group variations in network topological properties between patients with NMDAR‐resistant encephalitis and HCs. (A) The violin diagram depicts the AUC parameters of the Eglob, Eloc, and Lp for anti‐NMDAR encephalitis patients and HCs. Standard deviations and means are plotted. (B) Eglob, Eloc, and Lp across a density range between 10% and 34%. Each dot and error bar denotes the mean and standard deviation for every density threshold, individually. Asterisks signify a notable discrepancy at that density level. Abbreviations: AUC = area under curve, Eloc = local efficiency, Eglob = global efficiency, HCs = healthy controls, and Lp = shortest path length.

Compared to HCs, the AUC values of the network properties spotted a marked improvement over Eglob (t = 2.181, *p* = 0.032), Eloc (t = 2.253, *p* = 0.027), and decreased at Lp (t = −2.310, *p* = 0.022) in individuals diagnosed with anti‐NMDAR encephalitis (Figure 2).

**FIGURE 3:**
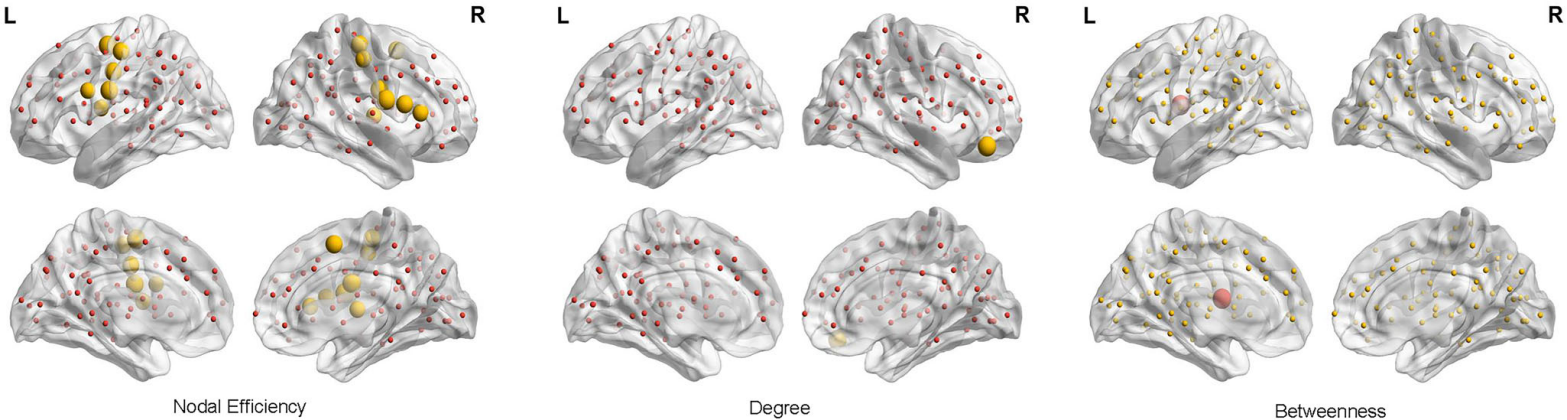
Group variations in nodal topological properties between anti‐NMDAR encephalitis patients and HCs. Group variations in efficiency, degree, and betweenness at the nodal level. Compared to HCs, the insignificant nodes of the anti‐NMDAR encephalitis patients are shown as small spheres, whereas bigger yellow (anti‐NMDAR encephalitis > HCs) and bigger red (anti‐NMDAR encephalitis < HCs) spheres show sizable variation. The magnitude of the significant nodes mirrors the effect sizes of group differences. **: *p* < 0.01. Abbreviations: anti‐NMDAR = anti‐N‐methyl‐D‐aspartate receptor, HCs = healthy controls.

For regional nodal features, the efficiency of lymph nodes had increased among anti‐NMDAR encephalitis patients in the DMN (ventral frontal cortex [t = 3.138, *p* = 0.003] and frontal lobe [t = 3.352, *p* = 0.001]), SMN (anterior central somatosensory‐motor network [t = 3.463, *p* = 0.001] and anterior central gyrus [t = 3.898, *p* < 0.001]), VAN (midbrain insula [t = 2.940, *p* = 0.005]), and an enhanced degree of lymph nodes in the DMN (left ventral medial prefrontal cortex [t = 3.524, *p* = 0.001]), compared with HCs. The intermediacy of SCN (thalamus [t = −4.067, *p* < 0.001]) lymph nodes of anti‐NMDAR encephalitis patients was decreased (Figure 3).

## 4 Discussion

We investigated the altered topological properties and FC at baseline in anti‐NMDAR encephalitis patients on the basis of a graph‐theoretic approach. We found that functional connectivity between most brain regions was reduced. Topological features consist of both network and regional lymph nodes. Compared to HCs, anti‐NMDAR encephalitis patients had improved local and global efficiency, meanwhile shorter shortest path lengths. These findings may indicate abnormal integration and segregation of functional brain networks in anti‐NMDAR encephalitis patients. For regional lymph nodes, anti‐NMDAR encephalitis patients had increased lymph node efficiency and degree in the DMN and increased lymph node efficiency and decreased intermediacy in the SCN compared to HCs.

Research on the whole organization of brain networks is grounded in graph theory (Bullmore and Sporns 2009; Bullmore and Bassett 2011). High local efficiency and clustering coefficients are symptoms of high‐level regional specialization of information processing in normal brain networks. Instead, high global efficiency and low path lengths denote a robust cerebral capacity to consolidate information (He et al. 2009; Reijmer et al. 2013). We found that the shortest path length was lower within anti‐NMDAR encephalitis patients than HCs, together with higher global and local efficiencies. Existing studies have demonstrated that the shorter Lp for a network would come with faster information transfer between nodes and higher global efficiency (Liu et al. 2020; Zhang et al. 2020). However, it is noteworthy that global efficiency does not automatically imply greater cognitive ability. Because the increase in global integration may mask the usefulness of critical knots and minimize network modularity. Therefore, it is hypothesized that the elevated global efficiency of anti‐NMDAR encephalitis patients is caused by their brain networks' tendency to develop randomly, which leads to a weakening of the role of key brain regions in anti‐NMDAR encephalitis patients (Guo et al. 2014; Li et al. 2017; Zhang et al. 2018). Nonetheless, some research reported changes in shortest path length and global efficiency in the individuals with anti‐NMDAR encephalitis that are inconsistent with those found in this paper (Luo et al. 2015), and these inconsistent results may be due to different ways of constructing brain networks, patient heterogeneity (disease duration, medication or not, sample size, and different stages of anti‐NMDAR encephalitis), and so on.

Impairment of DMN in anti‐NMDAR encephalitis patients has been demonstrated (Peer et al. 2017), including the posterior cingulate gyrus and medial prefrontal cortex (mPFC). Consistent with prior research, we further exemplified the role of DMN in anti‐NMDAR encephalitis. The mPFC principally engages in operating memory and executive functions (Colgin 2011; Miller and Cohen 2001), including task switching, inhibition control, and reactive repression (Chen et al. 2009; Rushworth et al. 2002; Yuan and Raz 2014), and it can avoid short‐sighted behaviors, reflect upon solutions to issues with their aftermath, perform self‐control, make decisions, and appropriately regulate emotions for long‐term goals. Neurons in and around the posterior cingulate gyrus sulcus stay crucial in the link between ruminative thoughts and pain (Koelsch et al. 2022). Prior research has shown extensive WM changes among anti‐NMDAR encephalitis patients, notably in the cingulate (Finke et al. 2013). We detected reduced FCs between the posterior cingulate gyrus and mPFC of anti‐NMDAR encephalitis patients, which may be associated with impaired emotion regulation. From a functional integration perspective, patients with anti‐NMDAR encephalitis have reduced connectivity between the sensorimotor cortex and the post‐DMN hub, suggesting decoupling of the SMN and DMN (Chen et al. 2023). The DMN focuses on self‐reflective awareness events (Chen et al. 2023), whereas the SMN engages with motor coordination and somatosensory processing. Diminished cross‐network integration between the SMN and DMN may scramble sensorimotor processing, causing reality distortion, which is implicated in positive symptoms of schizophrenia (Lee et al. 2018; Watsky et al. 2018). Thus, impaired connectivity between the posterior DMN and SMN in anti‐NMDAR encephalitis patients may lead to distorted self‐perception of internal and external sensory inputs, thereby contributing to the development of delusions and hallucinations.

The sensorimotor system helps to process abstract words and conceptual knowledge (Gallese and Lakoff 2005). The anterior central gyrus forms an integral part of the somatosensory‐motor network participating in modulation and somatosensory processing. It is primarily in charge of incorporating the information conveyed by diverse somatosensory stimuli to properly visualize objects. In contrast, the present study observed increased functional connectivity between specific brain regions within the somatosensory‐motor network (SMN)—namely, frontal lobe‐precentral gyrus and precentral gyrus‐parietal lobe—in patients with anti‐NMDAR encephalitis. A previous meta‐analysis revealed that reduced regional homogeneity of the SMN could explain psychomotor retardation (Iwabuchi et al. 2015). The terminal lobe serves vital physical features intimately tied to attention, perception, episodic recall, spatial cognition, and calculation (Corbetta and Shulman 2002; Sack et al. 2002). Meanwhile, as the neurological web theorization states, cognitive performance is not confined to a particular lobe or functional district of the brain; rather, there is a widespread and intense relationship between the diverse aspects. Strikingly, there is an intense functional connection between the hippocampus and the ventral parietal lobe in humans (Vincent et al. 2006), which is associated with cognition. In the present study, we observed reduced parietal‐anterior cingulate cortex (ACC) connectivity in patients with anti‐NMDAR encephalitis. Previous research has proposed that impaired functional connectivity between the insula and parietal lobules disrupts the feedback, transmission, and maintenance of information, leading to decreased cognitive function in patients (Li et al. 2021). We observed reduced parietal‐ACC connectivity in patients with NMDAR‐resistant encephalitis, which could explain the decline in cognitive scores. In addition, our study reports FC enhancement from DAN (posterior parietal) to SMN (parietal). It indicates that extensive functional network compromise occurs in anti‐NMDAR encephalitis, which is linked to the widespread distribution of NMDAR in numerous brain districts.

The occipital lobe (VN) is related to visual image processing and recognition, and the most coherent metabolic model in anti‐NMDAR encephalitis characterizing neuronal dysfunction is the phenomenon of occipital hypometabolism (Maeder‐Ingvar et al. 2011). Preceding investigations revealed that the dynamic coupling between VN, FPN, and DMN differs by mission objective (Chadick and Gazzaley 2011), and there was a significant reduction of FCs within VN (Peer et al. 2017). Our study found a decrease within the FC of patients between VN‐DMN, suggesting that this change may be linked to impairments of dealing with diverse information by patients.

Thalamus (SC) is a relay center for neural signal transmission procedures concerned with sensory and motor functions. Considerable proof suggests that the thalamus engages in emotion modification (Penzo and Gao 2021), and it serves an imperative part in emotions and staying alert, as well as additional cognition (Halassa and Kastner 2017). Previous evidence points to a bilateral thalamic gray matter volume in patients with anti‐NMDAR encephalitis exhibited smaller than in HCs (Long et al. 2022). Moreover, an MRI in a case report of a patient with anti‐NMDAR encephalitis displayed high bilateral thalamic densitometry with restriction of dissemination (Dubey et al. 2020). Previous studies have pointed to broad architectural linkages among the thalamus and hippocampus, prefrontal lobe, and other structures (Kafkas et al. 2020). The hippocampus is a core brain region involved in cognition, memory, and emotional processing, and previous studies have indicated that functional dysregulation in the hippocampal region is associated with signal transduction and neurogenesis (Sun et al. 2024). Our present study found a significant reduction in thalamic connections to other brain regions. We also observed lymph node features in the abnormal thalamus characterized by a decrease in centrality and an increase in efficiency of the lymph nodes in patients. This indicates that pathological damage and compensatory adaptation coexist in anti‐NMDAR encephalitis (Volz et al. 2016). Reduced FC and lymph node centrality in the thalamus could explain the emotional dysfunction seen in NMDAR encephalitis. Higher lymph node efficiency, which implies increased efficiency of thalamic coordination with other regions. Experiments in animals established that when localized brain functions got undermined, other sectors exerted a supplementary part to preserve normal functioning (Chen et al. 2021). Patients with anti‐NMDAR encephalitis typically show diminished memory capacity. Avoiding the consequences of these aggravating situations calls for triggering the compensatory mechanisms through increasing the efficiency of coordination between the thalamus and other regions.

Some restrictions exist within our research as well. First of all, the current research involved a rather small sample size considering the risk of multiple comparison bias, in future studies, we will further expand the sample size to improve statistical power, incorporate refined symptom assessment indicators (e.g., seizure frequency, positive and negative syndrome scale scores, and subscale scores of anxiety and depression scales), and conduct in‐depth verification of the quantitative associations between abnormal brain functional connectivity and specific clinical symptoms, thereby further addressing the gaps in current research. Secondly, with all patients undergoing various types of immunosuppressive medications, it would have been challenging to preclude the impact of medication efficacy on the outcome. Third, cross‐sectional design does not address causality. This study did not conduct longitudinal follow‐up, so it is unable to reveal the dynamic changes in brain functional connectivity. There is a need for future longitudinal studies to evaluate further topological and functional connectivity variations in the brains of anti‐NMDAR encephalitis patients.

## 5 Conclusion

We investigated abnormal brain topological features and altered FC networks in NMDAR patients by graph theory. This research supplies distinctive perspectives for learning about the brain dysfunction behind anti‐NMDAR encephalitis. Measured aberrations in the structure of the functional brain networks could assist in improved comprehension of neuropathologic and physiologic pathologies in patients with anti‐NMDAR encephalitis.

## Author Contributions

**Rui Qian**: writing – review and editing, validation, visualization, software, methodology, formal analysis. **Rong Guo**: writing – review and editing, writing – original draft, validation, software, formal analysis, investigation. **Yifei Li**: writing – review and editing, investigation, validation, formal analysis. **Chenglong Li**: writing – review and editing, validation, software, methodology. **Ling Wei**: writing – review and editing, validation, software. **Juanjuan Zhang**: writing – review and editing, validation, supervision. **Yuanyuan Guo**: writing – review and editing, validation, software, methodology, formal analysis. **Yanghua Tian**: writing – review and editing, validation, supervision, resources, project administration, methodology, funding acquisition, formal analysis, data curation, conceptualization.

## Data Availability

All data originating or consumed within the study can be requested from the corresponding authors.
